# Navigating the nano-bio immune interface: advancements and challenges in CNS nanotherapeutics

**DOI:** 10.3389/fimmu.2024.1447567

**Published:** 2024-11-12

**Authors:** Chantalle Moulton, Anna Baroni, Erica Quagliarini, Lucia Leone, Luca Digiacomo, Marta Morotti, Giulio Caracciolo, Maria Vittoria Podda, Ennio Tasciotti

**Affiliations:** ^1^ Human Longevity Program, IRCCS San Raffaele Roma, Rome, Italy; ^2^ Department of Molecular Medicine, Sapienza University of Rome, Rome, Italy; ^3^ Department of Neuroscience, Università Cattolica del Sacro Cuore, Rome, Italy; ^4^ Fondazione Policlinico Universitario A. Gemelli IRCCS, Rome, Italy; ^5^ Department of Human Sciences and Quality of Life Promotion, Università telematica San Raffaele, Rome, Italy

**Keywords:** nanoparticles, central nervous system, immunosurveillance, immune reprogramming, biomolecular corona, nanotherapeutics, molecular rehabilitation

## Abstract

In recent years, significant advancements have been made in utilizing nanoparticles (NPs) to modulate immune responses within the central nervous system (CNS), offering new opportunities for nanotherapeutic interventions in neurological disorders. NPs can serve as carriers for immunomodulatory agents or platforms for delivering nucleic acid-based therapeutics to regulate gene expression and modulate immune responses. Several studies have demonstrated the efficacy of NP-mediated immune modulation in preclinical models of neurological diseases, including multiple sclerosis, stroke, Alzheimer’s disease, and Parkinson’s disease. While challenges remain, advancements in NPs engineering and design have led to the development of NPs using diverse strategies to overcome these challenges. The nano-bio interface with the immune system is key in the conceptualization of NPs to efficiently act as nanotherapeutics in the CNS. The biomolecular corona plays a pivotal role in dictating NPs behavior and immune recognition within the CNS, giving researchers the opportunity to optimize NPs design and surface modifications to minimize immunogenicity and enhance biocompatibility. Here, we review how NPs interact with the CNS immune system, focusing on immunosurveillance of NPs, NP-induced immune reprogramming and the impact of the biomolecular corona on NPs behavior in CNS immune responses. The integration of NPs into CNS nanotherapeutics offers promising opportunities for addressing the complex challenges of acute and chronic neurological conditions and pathologies, also in the context of preventive and rehabilitative medicine. By harnessing the nano-bio immune interface and understanding the significance of the biomolecular corona, researchers can develop targeted, safe, and effective nanotherapeutic interventions for a wide range of CNS disorders to improve treatment and rehabilitation. These advancements have the potential to revolutionize the treatment landscape of neurological diseases, offering promising solutions for improved patient care and quality of life in the future.

## 1 Introduction

The central nervous system (CNS) has traditionally been considered a privileged immune system due to the presence of the blood-brain barrier (BBB) and the absence of traditional lymphatic drainage (1). However, it is now recognized that the CNS harbors a complex immune system comprising resident microglia, astrocytes, and infiltrating immune cells (2). These cells play essential roles in immune surveillance, neuroprotection, and neuroinflammation, influencing both physiological and pathological processes in the brain (3). Understanding the neuroimmune system is critical, as it underlies various physiological processes and plays a central role in health and disease (4).

Nanotherapeutics, the application of nanotechnology in therapeutic interventions, has emerged as a promising approach for addressing the complexities of treating CNS disorders (5, 6). The unique challenges inherent in delivering therapeutic agents to the CNS, such as the BBB’s selective permeability and the delicate microenvironment of neural tissues, have long hindered traditional drug delivery methods (7). Nanoparticles (NPs), with their customizable properties and nanoscale dimensions, offer a solution to this challenge (8). Engineered to carry therapeutic payloads across biological barriers and precisely target diseased cells or tissues, NPs hold great potential for revolutionizing CNS therapeutics (8). Their ability to navigate through the BBB, achieve sustained drug release, and minimize systemic side effects presents a paradigm shift in the treatment of CNS disorders (8, 9).

Understanding the intricate interplay between the immune system and NPs is paramount for the development of effective nanotherapeutic interventions (7, 8). The immune system in the CNS presents both challenges and opportunities for tailored nanotherapeutic approaches that account for the unique immunological landscape of the CNS (6, 7, 10). By elucidating the intricacies of immune responses within the CNS and their implications for NPs interactions at the nano-bio interface, it is possible to develop strategies aiming at enhancing drug delivery efficiency, minimizing immune-mediated clearance, and modulating immune responses for therapeutic benefit (7).

The nano-bio interface in the CNS is critical for developing NP engineering strategies that exploit or evade the immune system to enact therapeutic benefits (11, 12). Personalized NP engineering offers promise in precision medicine for CNS disorders, enabling targeted drug delivery, immunomodulation, and neural repair (13). By exploiting immune cell targeting pathways, NPs can selectively deliver therapeutic agents to diseased CNS tissues, enhancing therapeutic efficacy (14). Biomimetic NPs, mimicking natural extracellular vesicles like exosomes, can evade immune surveillance, facilitating efficient delivery of therapeutic payloads within the CNS (14). Studies have shown that NPs designed to evade immune detection or specifically target immune cells can significantly alter the inflammatory landscape in the CNS, impacting both therapeutic outcomes and potential side effects (15–20).

**Figure 1:**
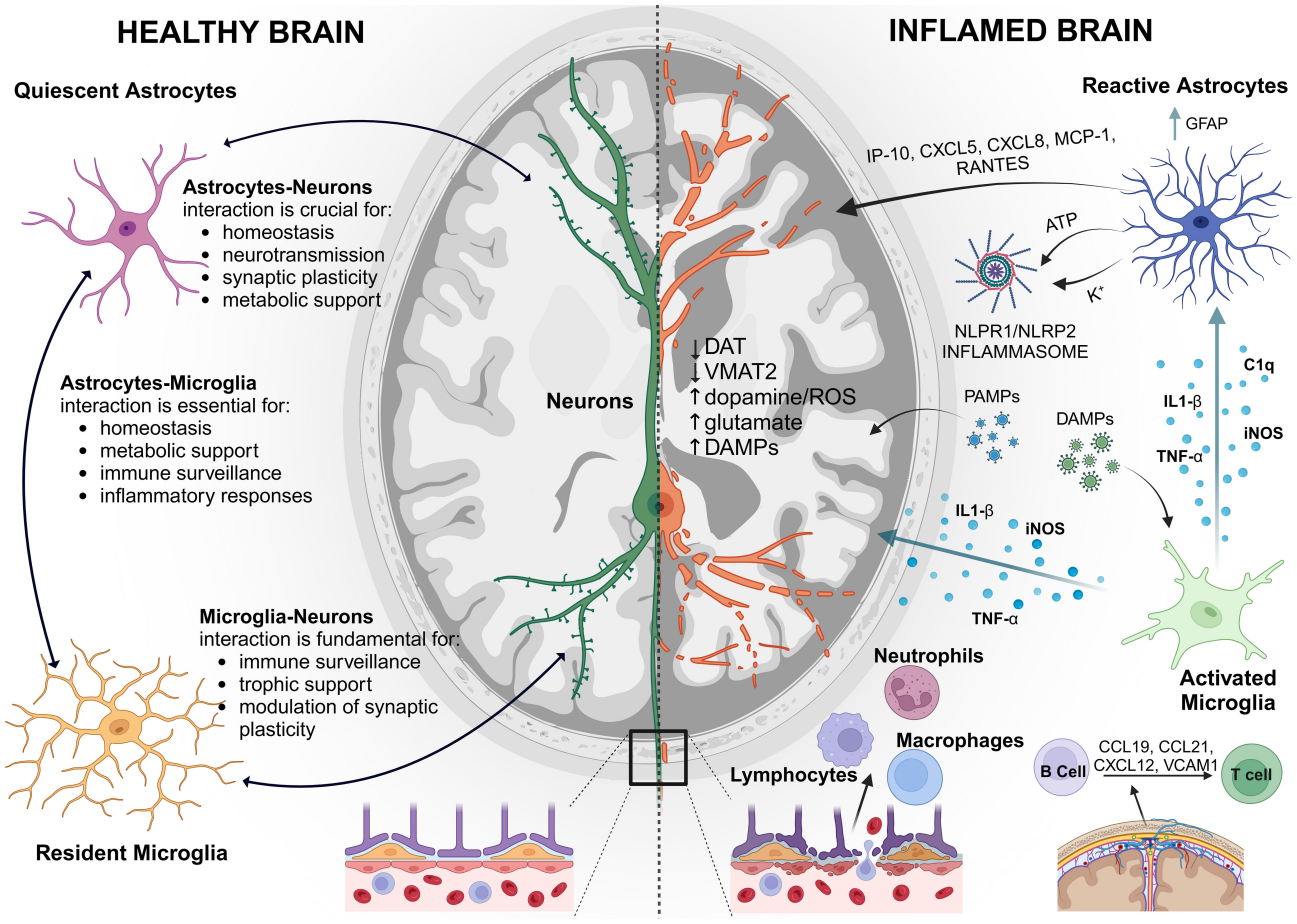
Schematic representation of the Neuroimmune System: On the left, a healthy brain with balanced immune activity. On the right, an “inflamed brain” showing activated microglia and astrocytes and exacerbated immune response, highlighting the brain’s defense mechanisms against injury and disease. GFAP, glial fibrillary acidic protein; IP-10, interferon-c-Inducible Protein; MCP-1, Monocyte chemotactic protein 1; K^+^, potassium ion; DAT, dopamine transporter; VMAT1, vesicular monoamine transporter type 1; VMAT2, vesicular monoamine transporter type 2; ROS, reactive oxygen species; C1q, complement component subunit 1q; iNOS, inducible nitric oxide synthase; TNF-α, tumor necrosis factor-α; IL-1β, interleukin-1 β; NLRP1/NLRP2, NOD-like receptors. Created with BioRender.com.

Within this frame, we summarized the key pathways involved in brain immune system activation emphasizing the crosstalk among different cell types (**Figure 1**). Additionally, we examine the dynamic interactions between NPs and the CNS immune system, including how NP properties influence immune recognition and surveillance and immune system reprogramming. We further investigate the formation and impact of the biomolecular corona of NPs, assessing its effects on NP behavior, and the nano-bio interface in the immune responses within the CNS. Through these objectives, we provide the latest insights that are more crucial for developing effective nanotherapies for the CNS.

## 2 Nanoparticles and the immune system in the CNS

### 2.1 The immune context of the CNS

#### 2.1.1 The blood-brain barrier and cellular constituents of the CNS immune system

The BBB is a dynamic and complex interface that separates the circulating blood from the brain parenchyma and regulates the exchange of molecules between the bloodstream and the brain. At its core are the brain microvascular endothelial cells, which form the primary barrier interface. Surrounding the endothelial cells are pericytes, which provide structural support and regulate blood flow. Astrocytic endfeet ensheath the microvessels and release signaling molecules that modulate BBB integrity and function. Additionally, the basement membrane, composed of extracellular matrix proteins, provides structural support and contributes to barrier properties (21).

A variety of cellular and molecular mechanisms adapt BBB to changing physiological conditions and protect the brain from insults. Crosstalk between the BBB and neuroimmune system plays a crucial role in orchestrating immune responses within the CNS and regulating BBB integrity under physiological and pathological conditions (22). In response to immune challenges or neuroinflammation, the BBB undergoes dynamic alterations, facilitating immune cell recruitment and cytokine signaling. Microglia, the resident immune cells of the CNS, survey brain parenchyma, monitoring for signs of injury, infection, or aberrant neuronal activity. Microglia exhibit a spectrum of activation states, ranging from surveillant to pro-inflammatory or anti-inflammatory phenotypes, depending on the microenvironmental cues (23). Astrocytes, traditionally viewed as support cells, also contribute to CNS immunity by releasing cytokines, chemokines, and growth factors in response to immune stimuli (24). In addition to resident cells, the CNS immune system includes infiltrating immune cells, such as T cells, B cells, and monocytes, which can enter the CNS during inflammatory or pathological conditions (25).

On the other side, immune mediators released by activated microglia or astrocytes can modulate BBB permeability and endothelial function, affecting CNS homeostasis (26). Dysfunction of the BBB has been implicated in the pathogenesis of various neurological disorders, including neurodegenerative diseases, cerebrovascular diseases and neuroinflammatory disorders. In Alzheimer’s disease (AD), disruption of the BBB leads to the accumulation of amyloid-beta plaques and neuroinflammation, contributing to disease progression (27). In stroke, ischemic injury and inflammation compromise BBB integrity, exacerbating neuronal damage and edema formation. In multiple sclerosis, immune-mediated damage to the BBB allows infiltration of autoreactive lymphocytes into the CNS, leading to demyelination and neurodegeneration (28). In neuroinflammation induced by low-dose lipopolysaccharide crosstalk between neutrophils and microglia occurs, through the brain blood vessel (29).

#### 2.1.2 Microglia: the resident immune sentinels

Microglia are the resident immune cells of the CNS, comprising approximately 5-10% of the total glial cell population. Historically viewed as passive responders to injury and infection, microglia are now recognized as dynamic and multifunctional cells that actively survey the CNS microenvironment and respond to various stimuli. In addition to their immune surveillance role, microglia actively participate in synaptic pruning, neurogenesis, and synaptic plasticity, contributing to the refinement of neuronal circuits during development and adulthood (30, 31). Microglia exhibit a ramified morphology characterized by a small soma and highly dynamic processes that continuously survey the surrounding microenvironment. Under homeostatic conditions, microglia establish a non-overlapping territory within the CNS, allowing them to efficiently monitor their local environment for signs of damage, infection, or aberrant neuronal activity. Under pathological conditions, microglia undergo activation and morphological changes, retracting their processes and adopting an amoeboid-like shape to migrate towards the site of injury or inflammation (32). Microglia express a diverse array of pattern recognition receptors (PRRs), including Toll-like receptors (TLRs), NOD-like receptors (NLRs), and RIG-I-like receptors (RLRs), which recognize pathogen-associated molecular patterns (PAMPs) or damage-associated molecular patterns (DAMPs) (33). Upon ligand binding, these receptors trigger intracellular signaling cascades, leading to the activation of transcription factors such as nuclear factor kappa B (NF-κB) and interferon regulatory factors (IRFs), and the subsequent production of pro-inflammatory cytokines, chemokines, and reactive oxygen species (ROS) (34). The NF-κB-pathway is a central regulator of inflammatory gene expression, promoting the transcription of pro-inflammatory cytokines, such as tumor necrosis factor-α (TNF-α), interleukin-1 β (IL-1β) and inducible nitric oxide synthase (iNOS). Similarly, the mitogen-activated protein kinase (MAPK) pathway, including ERK, JNK and p38 MAPK, mediates microglial activation and cytokine production in response to extracellular stimuli (35). Moreover, microglial inflammasome activation has been implicated in the pathogenesis of neuroinflammatory diseases, contributing to neurodegeneration and neuronal dysfunction. Dysregulated inflammasome signaling may result from mitochondrial dysfunction, ROS generation, or the accumulation of misfolded proteins (36).

Dysregulated microglial activation is also a hallmark of various neurological disorders, including AD, Parkinson’s disease (PD), and multiple sclerosis, where sustained neuroinflammation exacerbates neuronal damage and contributes to disease progression (37, 38).

#### 2.1.3 Astrocytes: guardians of CNS homeostasis

Astrocytes, the most abundant glial cells in the CNS are involved in several aspects of brain physiology and pathology, given their prominent role in homeostatic control of brain milieu (39). As such, although they are not considered immune cells, their role in the inflammatory response is well recognized.

Reactive astrogliosis is generally associated with microglial reactivity and leukocyte recruitment, under several pathological conditions characterized by neuroinflammation, including stroke (40), AD (41), PD (42) and brain senescence (43).

Reactive astrogliosis is a complex process which, depending on the severity of the injury, includes variable changes in gene expression, increased cellular proliferation and hypertrophy, and in severe cases leads to scar formation; the process initially can gain many beneficial functions in order to support neural tissue recovery, restore brain homeostasis, and limit tissue damage, but it can also have detrimental effects on neuron survival and axon regeneration.

Increased expression of glial fibrillary acidic protein (GFAP) and hypertrophy are prominent features of reactive astrocytes, yet not sufficient to unequivocally define the reactive state or phenotype. Seemingly, the dichotomy “A1” as neurotoxic and “A2” as neuroprotective phenotype has been gradually replaced by a more complex and dynamic feature, which implies molecular expression patterns and functional changes that might be even different depending on brain region and pathology (44).

In the context of neuroinflammation, activated microglia secrete pro-inflammatory mediators among which, TNF-α, IL-1α and complement component subunit 1q (C1q), which have been involved in astrocytes activation through the NF-κB signaling pathway, upregulating wide array of inflammatory response genes, with a crucial role attributed to complement component 3 (C3) (45, 46). A major pro-inflammatory pathway that seems to be activated in microglia-primed reactive astrocytes, involves the phosphorylation of IκBα promoting its dissociation from its complex with p50 and p65 allowing their phosphorylation and translocation from the cytosol to the nucleus to influence expression pro-inflammatory transcripts (47). Indeed, secretory astrocytes release pro-inflammatory chemokines such as interferon-c-Inducible Protein (IP)-10, CXCL5, CXCL8, Monocyte chemotactic protein1 (MCP-1), GRO-a, and RANTES, and cytokines such as IL-6, IL-1β, and TNF-α, granulocyte and macrophage colony stimulating factor (GMCSF), which, in turn, reinforce microglia to remain in an activated state in the context of bi-directional pro-inflammatory communication (48).

It has been demonstrated that also astrocytes express PRRs and other receptors involved in the detection of DAMPs, released by injured tissue, making them capable of detecting signals of brain damage (49), as canonical effectors cells of innate immunity. It is known that the activation of the different PRRs leads to transcriptional (cytokines and interferon genes) and non-transcriptional innate immune responses such as the induction of phagocytosis, autophagy, cell death, and cytokine processing (50). Specifically, astrocytes express an inflammasome that comprises the PRR, NOD-like receptors NLRP1/NALP2, the adaptor protein ASC and caspase-1 (51), which is necessary for enzymatic cleavage and maturation of the precursor cytokines pro-IL-1β and pro-IL-18 (52).

A model of inflammasome activation in the CNS has been proposed with ATP being released from dying cells after and activating P2X4. Once activated P2X4 would release potassium causing the opening of the pannexin-1 channel in neurons and astrocytes, leading to further release of ATP, contributing to the opening of the P2X7 receptor and subsequent activation of inflammasomes (49, 53).

The continuous research uncovering the mechanism of astrocyte activation in the complex landscape of neuroinflammation is of the utmost importance to pave the way for novel therapeutic approaches designed against astrocyte-specific targets.

#### 2.1.4 Neurons: orchestrators of immune responses

Traditionally viewed as the primary mediators of electrical signaling in the nervous system, neurons are now recognized also as active participants in immune regulation within the CNS. Neurons express a diverse array of immune-related molecules, including cytokine receptors, PRRs, and major histocompatibility complex (MHC) proteins, enabling them to detect and respond to immune signals (54). For instance, neurons can sense the presence of pro-inflammatory cytokines released by activated microglia and astrocytes, triggering intracellular signaling cascades that modulate neuronal excitability and synaptic transmission (55). Additionally, the expression of MHC class I molecules on neurons allows them to present antigens to cytotoxic T cells, thereby participating in immune surveillance and immune-mediated neuroprotection (56).

Another important aspect of the neurons is the profound effects on glial cell function, influencing their activation states and inflammatory responses. The recruitment and activation of glial cells during neuroinflammation are intricately regulated by the release of neurotransmitters from neurons, such as glutamate, GABA, and extracellular nucleotides like ATP and UDP (57). In fact, GABA and glycine have been shown to decrease the secretion of several cytokines induced by lipopolysaccharide and to attenuate the phagocytic activity of microglia (58). Glutamate release from neurons can exert dual effects on microglial activation; it can either inhibit microglia activation via metabotropic receptors or mediate glial activation through AMPA and kainate ionotropic receptors (59). Furthermore, activation of α7 nicotinic acetylcholine receptors has been shown to downregulate the release of pro-inflammatory cytokines such as IL-6 and TNF-α (60). Additionally, it promotes the increased expression of glial cell-derived neurotrophic factor (GDNF) (61) and enhances glutamate uptake via the glutamate/aspartate transporter (GLAST) (62), suggesting a neuroprotective role for acetylcholine through the inhibition of microglia. Acetylcholine has been also found to elevate intracellular Ca^2+^ levels, thereby triggering glutamate release, which subsequently modulates GABAergic transmission in astrocytes (63), influencing network oscillations. Moreover, cholinergic transmission impacts satellite glia, enabling them to support and enwrap sensory peripheral neurons that were previously lacking nerve growth factor (64). In turn, glial cells can modulate neuronal excitability and synaptic transmission through the release of inflammatory mediators, highlighting the dynamic interplay between neurons and glia in CNS immune regulation (65). Dysregulation of neuronal-immune interactions has been implicated in the pathogenesis of neuroinflammatory diseases, including multiple sclerosis (MS), AD, and PD (66).

#### 2.1.5 Peripheral immune cells: infiltrators and regulators. Leukocyte movement, surveillance, and penetration of the CNS macrophages and other myeloid cells

The migration of myeloid cells into the CNS occurs primarily in the context of injury or inflammation. Monocyte-derived cells are among the most abundant infiltrators in murine models of neuroinflammation, with their number correlating with axonal damage also in the brain biopsy of multiple sclerosis patients. These cells enter the CNS by rolling along activated endothelium, firmly adhering to cerebral vessels, then migrating into the subarachnoid space and parenchyma at sites of damage or neuroinflammation. Upon entering the subarachnoid space, activated monocytes differentiate into macrophages, presenting antigens to T cells, which in turn secrete cytokines, further activating brain inflammation. The phenotype of infiltrating macrophages evolves during neuroinflammation, transitioning from an inflammatory phase to a resolution phase. Immune surveillance of the brain by these monocytes is proposed to involve integrin LFA-1 for rolling and chemokines CCR2 and CX3CR1 for rapid tissue invasion (67). Mice lacking MCP-1, a ligand for CCR2, showed significantly reduced inflammation in EAE. CX3CR1 is vital not only for monocyte and myeloid cell trafficking into the CNS but also highly expressed on natural killer (NK) cells and lymphocytes entering the CNS. However, little is known about myeloid cell entry sites into the CNS, their source, and the mechanisms establishing residency at homeostasis (68).

Recent studies reveal that many monocytes and neutrophils residing in the brain and spinal cord originate not from blood but from CNS-associated bone marrow. These cells migrate from cranial bone marrow to meninges through microscopic vascular channels. Their differentiation resulted from local populations and not from peripheral tissue progenitors. CNS-associated bone marrow-derived monocytes exhibit fewer inflammatory traits compared to blood-derived ones, suggesting intrinsic differences and specialization for CNS function (69).

##### 2.1.5.1 B Cells

The significance of B cell interaction in neuroinflammatory diseases is evident in successful B cell depletion therapy for treating CNS diseases (70). In MS the number of B cells increases in CNS compartments with evidence of B cell migration and ectopic lymphoid tissue formation via the CXCL13-CXCR5 axis (71). The CXCL13 expression in intrameningeal follicles, active lesions, and cerebrospinal fluid (CSF) attracts CXCR5-expressing B cells to form ectopic lymphoid follicles. Other chemokines like CCL19, CCL21, CXCL12, and adhesion molecules VCAM1 are induced during inflammation, aiding B cell trafficking into ectopic lymphoid tissues. The B cells reactivate auto-reactive T cells by cytokine secretion or act as antigen-presenting cells to induce T cell differentiation into pathological condition. Abnormal cytokine production by B cells from MS patients suggests their immunomodulatory role via cytokine signaling.

Less is known about B cell trafficking into non-diseased brain and meninges. Recent studies indicate that meningeal B cells in mice primarily derive from local bone marrow and minimally from blood. Specialized vascular channels facilitate their migration from cranial bone marrow to meninges. Dura B cells exhibit tissue-resident characteristics, with evidence of response to neuroinflammation. Gut microbiota influences autoreactive B and T cell recruitment in EAE, suggesting a gut-brain axis in CNS inflammatory disease regulation (72).

##### 2.1.5.2 T Cells

Tissue-resident T cells are found in meninges, CSF, and parenchyma in humans and rodents. These T cells exhibit unique transcriptional profiles and increased functional capacity for certain cytokines, influencing neurological function. CNS T cells contribute to CNS homeostasis and can modulate neurotransmitter precursor availability. Meningeal T cells, for instance, affect social behavior and memory formation via cytokine signaling. CD4 and CD8 T cells in the brain express tissue-resident markers and cytokines, with potential roles in modulating synaptic plasticity and social behavior (73). Human parenchymal T cells also display a tissue-resident phenotype, with implications for CNS immunity and neuroregulation (74). The interactions between CNS T cells and microglia are vital for neurodevelopment and homeostasis, although mechanisms remain unclear.

### 2.2 Nanoparticles in the immune nano-bio interface

#### 2.2.1 Nanoparticles

NPs are synthetic structures that possess unique physical and chemical properties due to their high surface area to volume ratio (75). Recently, NPs have become a significant trend in drug delivery, particularly as nanotherapeutics, due to their ability to enhance the solubility, stability, and bioavailability of drugs. They offer targeted delivery, controlled release, and reduced side effects, making them ideal for treating various diseases. NPs show great promise for treating CNS disorders due to their ability to cross the BBB, a major obstacle in delivering therapeutics to the brain (9, 76, 77). The BBB restricts the entry of various large molecules and therapeutic agents into the brain, but NPs can traverse this barrier through various mechanisms, including by passive transport, such as paracellular pathways between tight junctions and transcellular pathways, and by active transport, such as carrier-mediated transcytosis, receptor-mediated transcytosis, adsorptive mediated transcytosis and disruptive mediated transport (78). This capability offers potential breakthroughs in treating conditions such as AD, PD, and brain tumors (9, 76, 77). By successfully crossing the BBB, NPs can reach the CNS and interact with the neuroimmune environment, to improve rehabilitation and treatment of various acute and chronic CNS conditions to restore proper function.

Many types of organic and inorganic materials can be employed to construct NPs for medical applications, including lipids, polymers, metals and carbon according to the specific applications (75). Synthetic NPs include metallic and polymeric varieties. Polymeric NPs, for example, offer versatility in both design and functionality. Protein-based NPs, such as those constructed from ferritin, albumin, or collagen, can be engineered to display surface ligands that specifically target and interact with immune cells (79). Metallic NPs, such as those based on gold or silver, possess unique optical and physicochemical properties, making them useful in drug delivery and diagnostic applications (75, 80).

On the other hand, biomimetic NPs, such as lipid-based liposomes, cell-membrane-derived, and ghost NPs, mimic natural biological structures (81, 82). Extracellular vesicles (EVs) are natural lipid vesicles secreted by cells into the extracellular space (83, 84). There are three major types of EVs, including exosomes, microvesicles, and apoptotic bodies, which are distinguished based upon their biogenesis, release mechanisms, size, and function (83–85). Among these membrane vesicles, the role of exosomes in theragnostic research has been rapidly growing over the last two decades due to their small size (between 30 and 150 nm), their biocompatibility and inherent targeting capabilities. Inspired by these natural structures, biomimetic NPs offer unique advantages for immunomodulation (81, 86, 87). For example, lipid-based NPs, including liposomes, are capable of encapsulating and delivering various immunomodulatory agents, such as cytokines, antibodies, or small interfering RNAs (88, 89).

Furthermore, cell-derived NPs can be derived from the membranes of various cell types, such as immune cells, stem cells, or even cancer cells (90). They can carry specific surface proteins and signaling molecules that can interact with and modulate the function of immune cells. By incorporating biomimetic cues and surface modifications, polymeric NPs can target specific immune cells and modulate their activity (75, 91). Another class of biomimetic NPs includes Nano-Ghosts, which are technologically reconstructed using the cytoplasmic membranes, such as of mesenchymal stem cells, and are able to maintain the orientation composition, and function of the mesenchymal stem cell membrane as an inert particle. Notably, these Nano-Ghosts maintain the ability to selectively target and penetrate tissues of interest while being quickly cleared from other organs, with minimal off-target effects (92–95).

#### 2.2.2 Immunosurveillance of nanoparticles in the CNS

##### 2.2.2.1 Immunosurveillance of nanoparticles

**Figure 2:**
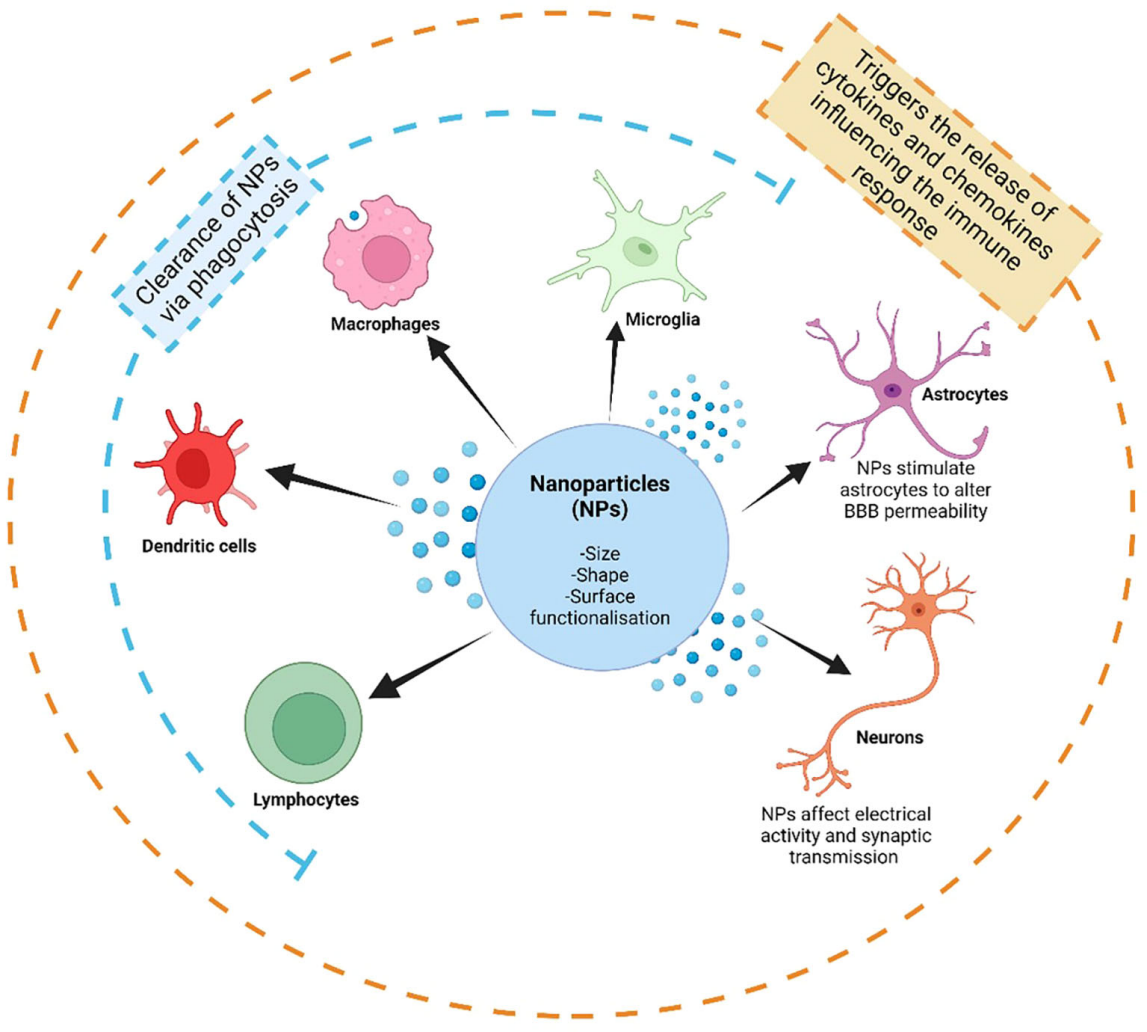
The immunosurveillance of nanoparticles (NPs) by the immune system in the central nervous system (CNS). The immune microenvironment in the CNS is dictated by microglia, the resident immune cell in the CNS, and interactions with astrocytes and neurons, as well as by infiltrating peripheral immune cells, including lymphocytes (such as T-cells and B-cells), dendritic cells and macrophages. The interactions between NPs and the immune system are influenced by the size, shape and surface functionalization and modifications of NPs. These interactions may cause the clearance of NPs via phagocytosis by certain immune cell types, as well as by stimulating many cell types to trigger further interactions, such as the release of cytokines and chemokines. These interactions, especially with astrocytes, may affect the regulation of blood brain barrier (BBB) permeability, and in turn further influencing the amount of infiltrating peripheral immune cells. Created with BioRender.com.

In the CNS, NPs encounter a sophisticated immunosurveillance system involving all the immune cells previously described (**Figure 2**) (10, 96). Microglia, as the resident immune cells of the CNS, play a crucial role in monitoring the brain environment for foreign substances, including NPs, through pattern recognition receptors like Toll-like receptors (TLRs) (97). Upon NP entry into the CNS, microglia can recognize and phagocytose NPs, influencing their clearance and distribution within the CNS (97). Astrocytes also contribute significantly to immunosurveillance by detecting NPs through various receptors and triggering cellular responses like cytokine release (45, 98). Neurons, although not traditional immune cells, interact with NPs, impacting neuronal health and function through the modulation of electrical activity and synaptic transmission (99, 100). Finally, infiltrating immune cells like macrophages, dendritic cells, and lymphocytes participate in the immunosurveillance of NPs within the CNS, recognizing NPs through receptors like TLRs and scavenger receptors (101).

The immunosurveillance of NPs in the CNS poses challenges to their efficiency, affecting their fate, biodistribution, and immunomodulatory effects (10, 96). Infiltrating immune cells like macrophages, dendritic cells, and lymphocytes can recognize NPs through various receptors, leading to phagocytosis and clearance, especially during inflammation or injury when the BBB becomes permeable (10, 102, 103). Phagocytosis, a process executed by immune cells like macrophages, neutrophils, and monocytes, is influenced by particle geometry, with anisotropic shapes resisting phagocytosis longer than spherical ones (104). The physicochemical properties of NPs, such as size, shape, and surface functionalization, play a crucial role in determining their interactions with immune cells and subsequent immunomodulatory effects (10, 105, 106).

Size and shape are important factors governing NP immunosurveillance (11). Smaller NPs tend to evade immune detection, and those smaller than 200 nm are considered theoretically small enough to cross the BBB (11, 107). Moreover, the shape of NPs has been shown to affect their interactions with the immune system (108–110). For example, gold nanorods are more efficiently internalized by macrophages than nanospheres due to the generation of large vesicles that facilitate entry through macropinocytosis (111). Moreover, nanorods, of aluminum oxyhydroxide nanoparticles, were found to activate the NLRP3 inflammasome, leading to IL-1β production in THP-1 cells and bone marrow-derived dendritic cells (112). Nanorods with higher aspect ratios also reduced phagocytosis and enhanced cytokine secretion, such as IL-6 and IFN-γ (113). Furthermore, it has been shown that while spherical and star-like gold NPs accumulated similarly in the liver, only the star-shaped NPs were able to localize in the lungs (114). Moreover, TiO_2_ microparticles adorned with nanospikes affects the interaction of the NPs with the innate immune system. During phagocytosis, the nanospikes exert mechanical stress on cell membranes, triggering potassium efflux and inflammasome activation in a caspase-1- and NLRP3-dependent manner. This mechanical stress, combined with TLR4 pathway activation, enhances dendritic cell maturation and significantly boosts both T-cell and humoral immune responses (109).

##### 2.2.2.2 Strategies to overcome NP sequestration by the immune system

As summarized in **Table 1**, multiple strategies have been developed in order to prevent NP sequestration by the immune system. Size, shape and surface optimization of NPs can minimize immune recognition and maximize tissue penetration, enabling efficient delivery of therapeutic payloads (142), either evading immune recognition and clearance, or prolonging circulation time and tissue accumulation within the CNS (143). Functionalizing NP surface with biocompatible polymers like polyethylene glycol (PEG) is widely regarded as the most effective strategy for protecting NPs from immune clearance and enhancing their bioavailability in the CNS (115). However, undesirable immune-related adverse effects have been linked to the presence of PEG on drug delivery vectors (144). For example, activation of the complement system, hypersensitivity reactions, and accelerated blood clearance (ABC) upon repeated administration are believed to be triggered by the generation of anti-PEG antibodies (116, 145). These antibodies subsequently facilitate the recognition of PEGylated NPs by the immune system (146).

**Table 1:** Summary of the strategies used to prevent nanoparticle (NP) sequestration by the immune system.

Strategy	Description	Mechanism	References
PEGylation	Coating NPs with polyethylene glycol (PEG) to reduce immune recognition and extend circulation time.	PEG creates a hydrophilic “stealth” layer around NPs, reducing protein adsorption (opsonization) and recognition by phagocytes, delaying clearance.	(115–118)
Surface Charge Modification	Engineering the surface charge of NPs to reduce immune activation and phagocytosis.	Neutral or zwitterionic charges reduce non-specific interactions with immune cells, while cationic NPs improve uptake by immune cells like macrophages, depending on the application.	(119–122)
Biomimetic Coating	Camouflaging NPs with natural cell membranes to mimic the body’s own cells and evade immune detection.	Biomimetic coatings allow NPs to mimic the surface properties of natural cells, reducing immune clearance and allowing them to circulate longer or target specific tissues.	(15, 123–125)
Decoy or “Backpack” Strategy	NPs attached to immune cells’ surfaces as “backpacks” to avoid phagocytosis and leverage immune cell transport.	Cellular backpacks prevent phagocytosis by inhibiting actin structure formation necessary for engulfment, allowing prolonged circulation and immune evasion while preserving cell functions.	(16, 17, 126–128)
Hitchhiking NPs	NPs attached directly to immune cell membranes, using the immune system’s transport mechanisms for CNS delivery and to evade detection.	NPs attach to immune cells via weak interactions (e.g., electrostatic, hydrophobic) or receptor-ligand recognition, allowing immune cells to transport them past immune surveillance barriers.	(18, 129–133)
Trojan Horse Strategy	Using phagocytic immune cells (e.g., macrophages or monocytes) to engulf and deliver NPs to target tissues, including the CNS.	Phagocytosis allows NPs to bypass immunosurveillance, leveraging receptor interactions, actin polymerization, and phagosome formation. Stem cells can also be used for targeted delivery to diseased tissues.	(20, 134, 135)
Other Hydrophilic Polymer Coatings	Using hydrophilic polymers (e.g., hyaluronic acid, polyoxazolines) to prevent protein adsorption and immune recognition.	Hydrophilic coatings create a water-rich layer around NPs, preventing opsonization and reducing phagocytic uptake, enhancing stability in biological environments.	(136–139)
Size and Shape Optimization	Adjusting NP size and shape to evade immune detection and enhance circulation time.	Ultrasmall NPs achieve innate immune invisibility, while rod-shaped or elongated NPs show prolonged circulation due to altered interactions with immune cells and tissues.	(109, 110, 119, 140, 141)

Of note, biomimetic NPs have attracted substantial interest for their potential to evade immune clearance, prolong circulation time, and improve targeting (82). NPs coated with cell membranes (CM-NPs) can disguise themselves as native cells, thus avoiding detection by the immune system (147). These nanocarriers exploit the natural properties of cell membranes combined with engineered cores. The first biomimetic CM-NPs were created using leukocytes coating of a silicon core (123, 124), or red blood cells (RBCs) membrane coating a poly(lactic-co-glycolic acid) (PLGA) core (14). This innovation greatly increased circulation time compared to PEG modification due to the natural identity provided by leukocytes and RBCs which reduced mononuclear phagocyte system sequestration. Since then, CM-NPs have been developed using a variety of cells, including platelets (148), white blood cells (149), cancer cells (150), bacteria (151), and neurons (87) each providing unique benefits. Additionally, these cell membranes have been functionalized with targeting ligands to improve delivery to specific sites, enhancing both effectiveness and safety, and have been explored as nanotherapeutic options in the CNS (15). This practice has further developed into the use of specific cell membrane proteins directly into the structure of NPs, to improve targeting in the CNS. Neuron targeting by NPs was improved in this manner, through the use of cell membrane proteins, such as from human pluripotent stem-cell-derived neurons to make humanized biomimetic nanovesicles for neuron targeting (87, 125, 152).

Other strategies have been developed whereby immune cells may act as carriers of NPs hitchhiking to the cells as “backpacks”, or hiding in the cytoplasm as Trojan horses (153) to avoid immunosurveillance in the CNS, and evade immune detection. Cellular backpacks, typically composed of polymer layers measuring up to 10 μm and exhibiting anisotropic shapes, can adhere to cells and avoid immediate phagocytic uptake (16, 17, 126, 127) because their design inhibits the formation of the actin structures necessary for phagocytosis (104). These backpacks comprise a cell-adhesive region, a payload region, and a release region, which degrades rapidly under specific conditions like low pH, exposing the payload directly to the cellular environment (128). Ensuring that the cell-adhesive region properly anchors the structure to the cell membrane is key to avoid impairing the cells’ functions, such as brain migration and immune regulation (126).

Hitchhiking NPs attach directly to the membranes of carrier cells, takes advantage of the immune system’s transport mechanisms to evade CNS immunosurveillance (18, 129). These NPs use receptor-ligand recognition, chemical bonding, or physical adhesion for attachment (129). Physical adhesion methods, including electrostatic interactions, hydrophobic interactions, van der Waals forces, and hydrogen bonding, require minimal modification (130–133). Although these weak interactions result in limited stability during circulation, the abundance of receptors on cell carriers provides a reliable attachment method. Ligand modifications on NPs can enable them to attach to various cell types, potentially increasing their utility as CNS delivery vehicles. However, the numerous receptors may lead to non-specific attachment, and receptor-ligand interactions could disrupt cell functions (129, 154). Cell surface proteins provide active groups, such as amines and thiols, for NP anchoring (155, 156).

The Trojan horse strategy can exploit the natural phagocytic ability of immune cells, particularly macrophages and monocytes, to deliver NPs to the CNS (134), as these cells are ideal candidates due to their significant phagocytic capacity (19), but has also been further developed using stem cells such as mesenchymal stromal cells (135). The process involves receptor interactions, cytoskeleton rearrangement, actin polymerization, and phagosome formation (20). NP properties, such as size, shape, surface chemistry, and mechanical characteristics, influence their uptake by macrophages. Hydrophobic NPs are more easily phagocytosed compared to hydrophilic ones, and cationic NPs are more readily engulfed due to the negative charge of macrophage membranes. This method utilizes the immune system’s natural processes to ensure efficient delivery of therapeutic agents to the CNS, bypassing typical immunosurveillance mechanisms.

Finally, NP size has the power to shield NPs from immune detection, where smaller NPs may prevent immune detection (140, 141). Zhu and colleagues (140) developed a size-tunable method of developing spherical ultra small gold NPs, of sizes ranging from 3 to 15nm, with significant benefits for scaling up production while minimizing batch variability. The ultra-small gold NPs showed excellent biocompatibility and immunocompatibility, with no observed toxicity to monocytes or macrophages. They also exhibited very low thrombogenicity and evaded detection by the complement system. Of note, ultrasmall NPs of roughly 2nm in size have been shown to cross the BBB (157, 158).

#### 2.2.3 Nanoparticle-induced reprogramming of the immune system

**Figure 3:**
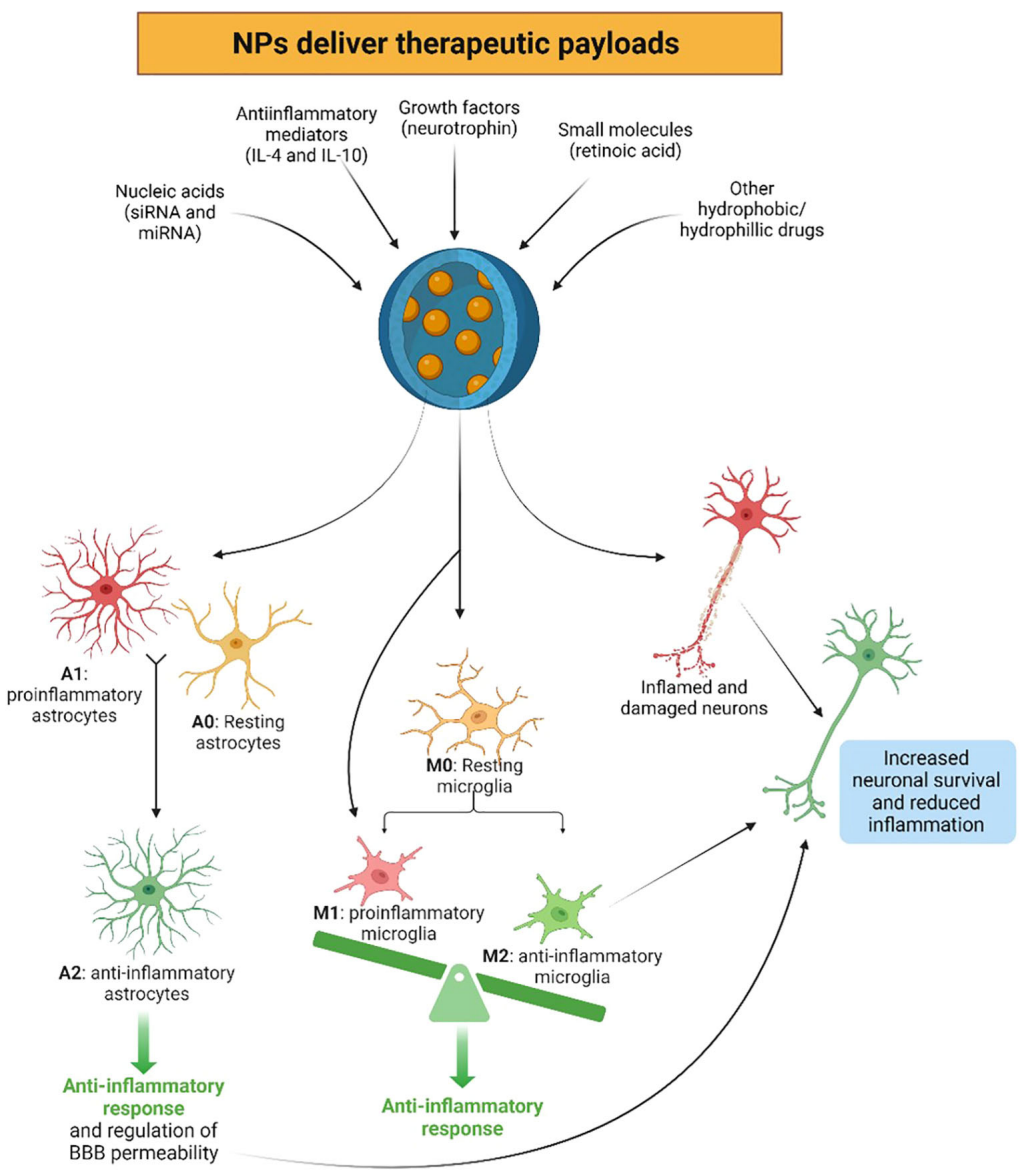
Nanoparticles (NPs) have the ability to beneficially reprogram the immune system in the central nervous system (CNS) by delivering therapeutic payloads, such as anti-inflammatory mediators, growth factors, nucleic acids, small molecules, as well as various hydrophobic and hydrophilic drugs. Due to NP uptake, resting (A0) or proinflammatory (A1) astrocytes may be reprogrammed to the anti-inflammatory and neuroprotective phenotype (A2), exerting anti-inflammatory responses in the CNS. Similarly, microglia can be programmed to the anti-inflammatory phenotype (M2) via the uptake of NPs, initiating anti-inflammatory responses, contrary to the resting (M0) and proinflammatory (M1) phenotypes. These NPs can also directly benefit neural survival, as well as through the beneficial anti-inflammatory effects exerted by the newly reprogrammed A2 astrocytes and M2 microglia. Created with BioRender.com.

NPs possess properties that enable precise interaction with immune cells, leading to immunomodulation and potential neuroprotection (14, 159). For example, NPs can reprogram immune cell populations within the CNS to induce beneficial shifts in immune function (**Figure 3**) either by delivering immunomodulatory agents or targeting specific immune cell receptors, which modulate immune responses and promote immune tolerance (105, 106).

As previously stated, one key mechanism involves the functionalization of NP surfaces with specific ligands to target receptors or antigens on immune cells such as microglia, astrocytes, and infiltrating lymphocytes. For example, modifying NPs with antibodies targeting microglial receptors like CD11b or TREM2 enables precise interaction, enabling much higher microglial internalization than control NPs, which resulted in the reduction of both pro-inflammatory cytokines and ROS in microglia, allowing for the rapid inhibition of microglial activation (14, 159, 160). Additionally, NPs can be engineered to carry molecules that alter the activation, proliferation, or cytokine production of immune cells, thereby shifting the immune response balance in the CNS (9, 105, 106). Also, NPs can induce immune tolerance, reducing autoimmune reactions or inflammatory responses (14, 159, 161). For instance, NPs engineered to release growth factors or anti-inflammatory mediators promote the repair and regeneration of damaged neural tissue (105, 162, 163). Furthermore, NPs can also serve as carriers for nucleic acids like siRNA or miRNA, which modulate the expression of immune regulatory genes, dampening pro-inflammatory responses and promoting immune tolerance (14, 159, 164), delivering therapeutic payloads directly into the cytoplasm of immune cells, influencing gene expression, protein synthesis, and cellular signaling pathways (105, 106).

Microglia, the resident immune cells of the CNS, are key targets for NPs in modulating neuroinflammation. NPs can reprogram microglial polarization from a pro-inflammatory (M1) to an anti-inflammatory (M2) phenotype, attenuating neuroinflammation and promoting tissue repair (105, 162). For instance, NPs loaded with anti-inflammatory cytokines such as IL-4 or IL-10 can polarize microglia towards an M2 phenotype (105, 159, 162). Retinoic acid-loaded NPs have shown effectiveness in reducing nitric oxide release and promoting the production of IL-4, enhancing neuronal survival and function (165). Moreover, astrocytes, which regulate CNS homeostasis and neuroinflammatory responses, can also be modulated by NPs. NPs loaded with neurotrophic factors or anti-inflammatory agents can attenuate astrocyte activation and promote neuroprotection in CNS diseases (105, 162).

One major challenge in using NPs for immune reprogramming in the CNS is their potential immunogenicity, which can trigger adverse immune reactions, inflammation, and tissue damage, compromising therapeutic efficacy (166). Another challenge is the risk of off-target effects, where NPs inadvertently modulate immune responses in unintended cell populations (167). To address these challenges, several strategies are employed. Functionalizing NPs to selectively target specific immune cells, such as microglia or infiltrating lymphocytes, can enhance specificity and reduce off-target effects (9, 168). Additionally, using NPs to encapsulate and deliver immunomodulatory agents or nucleic acids can exert precise control over immune cell activation, polarization, and cytokine production, which is crucial for addressing neuroinflammatory conditions, autoimmune disorders, and neurodegenerative diseases (77, 166).

Liposomes, with their aqueous core encased in a phospholipid bilayer, are versatile carriers for both hydrophobic and hydrophilic drugs (169). Currently, Phase III clinical trials are evaluating liposomes loaded with cytarabine for treating neoplastic meningitis. These liposomal NPs have demonstrated the ability to maintain therapeutic levels of cytarabine in the cerebrospinal fluid (CSF) for up to 14 days after administration (170). Another study utilized cationic nanoliposomes with transferrin receptor (TfR)-affinity ligands to deliver oligonucleotides and siRNA to the brain within six hours following intravenous injection. This approach effectively reduced neuroinflammation by targeting TNF-α with siRNA (171). Innovative NPs formulations are continually being developed for CNS applications. For instance, biodegradable PEGylated selenium NPs, conjugated with anti-TfR monoclonal antibody (OX26), have been shown to suppress pathological inflammation and oxidative metabolism linked to cerebral stroke (172). Additionally, inorganic gold NPs with diverse surface ligands hold potential for treating CNS bacterial infections due to their inherent bactericidal properties and the ability to conjugate antibiotics (80). These advancements highlight the ongoing efforts to exploit and evade the immune system for effective CNS drug delivery. By taking advantage of these strategies, researchers can develop targeted approaches to modulate immune responses within the CNS, offering new avenues for treating a wide range of neurological disorders.

Furthermore, nanozymes are NPs with catalytic properties that mimic natural enzymes, enabling biochemical reactions (173, 174). Platinum-based nanozymes (PtNZs) demonstrate effective catalytic activity, particularly in reducing inflammation through ROS scavenging (173). Studies have shown their biocompatibility and potential in treating inflammation, including neuroinflammatory conditions. For instance, PtNZs administered to mice following ischemic stroke were able to cross the damaged BBB, reduce matrix metalloproteinase-9 (MMP-9), and improve motor function. These particles show promise for future therapies targeting brain inflammation and neurodegenerative diseases (173, 175–178). Additionally, trimetallic nanozymes (PtPdMo-NZs), designed for enhanced catalytic efficiency, demonstrated high selectivity in neutral pH environments, further supporting their potential in treating chronic neuroinflammatory conditions like AD (173, 179, 180).

#### 2.2.4 The role of the biomolecular corona in the nano-bio immune interface

##### 2.2.4.1 Impact of the biomolecular corona on the nano-immune interactions

The nano-bio interface represents a pivotal junction to understand the intricate interplay between NPs and the biology of the CNS microenvironment (181). At this interface, NPs encounter a dynamic milieu characterized by the presence of various immune cells, soluble factors, and extracellular matrix components, all of which influence NPs behavior and outcome (182, 183). Understanding the nano-bio immune interface is fundamental since these complex interactions are the key to designing and optimizing therapeutic interventions targeting CNS disorders.

A widely recognized approach to enhance the efficacy of nanomedicine involves targeting NPs to specific sites for improved diagnosis or therapy. However, less than 1.0% of a given nanomedicine reaches this intent, leading to inefficient drug delivery and hampering clinical translation (184, 185). To tackle this issue, it is crucial to comprehend the interaction between NPs and the human body, particularly focusing on what is known as the “biomolecular corona (BC) effect”.

In fact, the original physical-chemical properties defining the *synthetic identity* of NPs undergo significant alterations upon exposure to biological environments. This is related to the spontaneous interaction between the biomolecules of the biofluids and NPs surfaces and the subsequent formation of a protein-enriched layer, indeed the BC (186). As a result, the formed NP-BC complexes acquire a distinct and novel biological identity leading to a reprogramming of the biomedical and physiological characteristics of the NPs.

Actually, BC represents the biological interface mediating NP-cell interactions, as it is involved in manifold biological processes and ultimately impacts on NPs’ biodistribution, targeting efficiency and immunogenicity (187). Furthermore, the BC can activate the immune system triggering inflammatory responses, NP clearance from the body, and cellular toxicity (188). Understanding the correlation between NP properties, BC formation, and its reaction at the nano-bio interface is crucial for monitoring the fate of the NP in biological compartments and predicting physiological responses. For the past decade, researchers have been diligently investigating the primary factors influencing the formation of the BC *in vivo*, with the underlying assumption that this understanding could facilitate the deliberate manipulation of BC formation and composition through strategic NP design (189, 190).

It has been discovered that BC equilibrium composition and structure is due to the interplay of shaping factors belonging to (i) the physicochemical properties of NPs (e.g., size, surface chemistry, shape, charge, aggregation after synthesis, (ii) environmental factors (e.g., incubation time, temperature, and shear stress), and (iii) the protein source and concentration (186, 191–193). Among these factors, the protein source (e.g., human vs. mouse serum) is the primary contributor to the enrichment of the BC with immunogenic proteins such as immunoglobulins and complement proteins (194). Variations in immunogenic protein profiles can markedly influence the pharmacokinetic behavior of NPs in the bloodstream, thereby casting doubt on the efficacy of animal testing in predicting physiological responses in humans. Furthermore, even disparate protein sources originating from the same animal species can elicit divergent responses to NP administration, as instance by triggering or mitigating the immune responses (191, 195). In fact, as the BC changes the inherent properties of NPs, it dictates how immune cells recognize and interact with the particle, both by non-specific and molecular recognition mechanisms.

**Figure 4:**
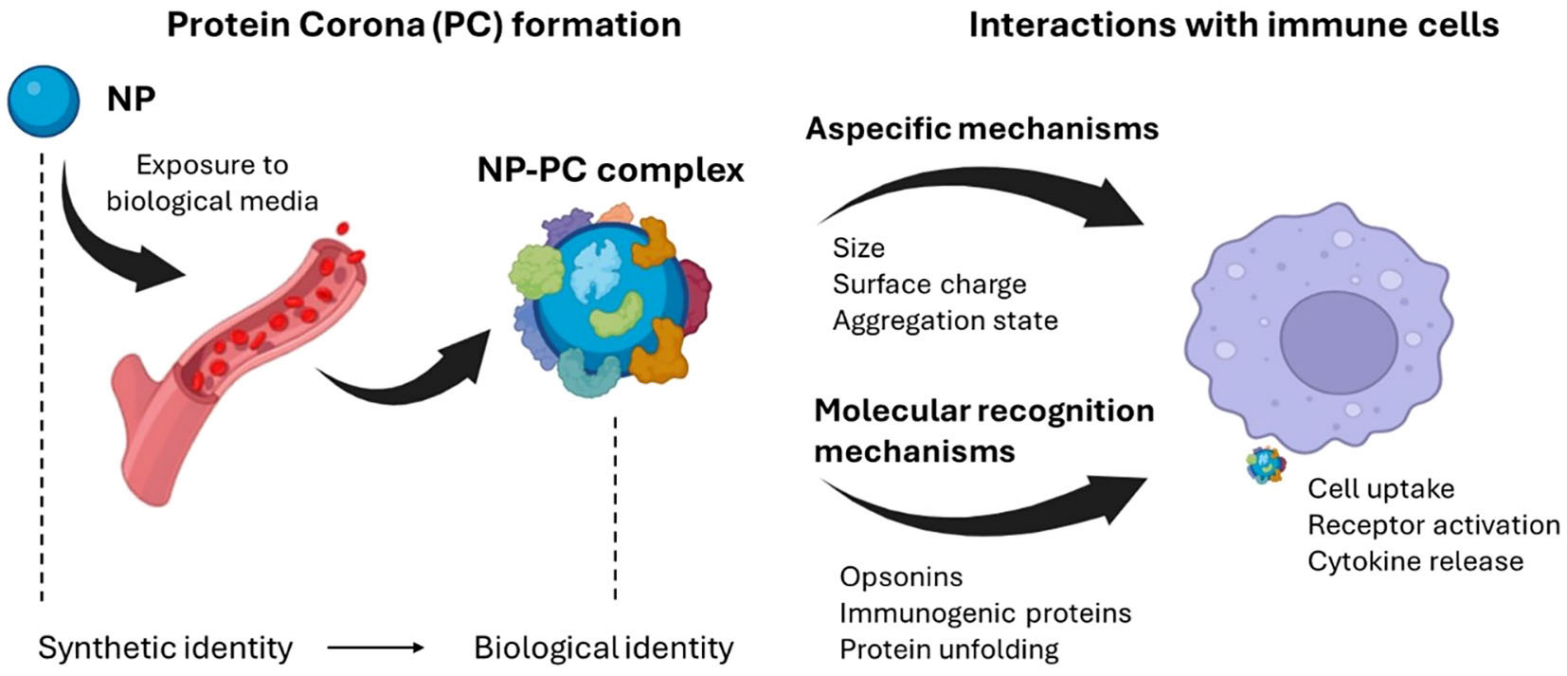
Exposure of nanomaterials to the biological environment alters the synthetic identity of the material, generating a protein-enriched layer known as the protein corona (PC). This new biological identity significantly impacts the physiological response, including the immune response. Among the biological effects induced by the PC is the activation of the immune system through nonspecific mechanisms, often leading to clearance from the circulatory system, or molecular recognition mechanisms. For instance, when the PC is enriched with specific proteins (e.g., opsonins), it may induce receptor-mediated inflammatory processes, degradation by phagocytes, or activation of the complement system. Created with BioRender.com.

At a first level, BC-induced alterations of NP chemical-physical features regulate non-specific processes of immune system activation (188). Those processes are not directly related to the BC composition but rather to a dynamic interplay of manifold factors, such as size, surface charge, and aggregation state of NP-BC complexes. Enlargement of NP size in biological environment is firstly due to the steric hindrance of the formed BC and depends on NP type. Indeed, BC thickness has been reported to range from about 20 nm [for 30–50 nm citrate-stabilized gold NPs (196)] to 35 nm [for 200 nm PSOSO_3_ NPs (197)]. As most plasma proteins have hydrodynamic diameters in the range of 3–15 nm, the measured values of BC thickness are compatible with a multi-layered structure, in which ‘primary binders’ recognize the nanomaterial surface directly, and ‘secondary binders’ associate with the primary binders via protein–protein interactions (198, 199). Of note, size increase of NPs is not only ascribable the steric hindrance of this multi-layered structure, but also to the formation of NP-BC aggregates (197, 200). This process occurs as the adsorbed proteins neutralize the surface charge of NPs, diminishing the mutual electrostatic repulsion and promoting the formation of short-range van der Waals bonds (188, 201). One of the main consequences of NP clustering in large aggregates is their immediate recognition by immune systems cells and the subsequent clearance from the bloodstream, principally by splenic filtration and phagocytosis by mononuclear phagocyte system (MPS) in the liver (202). Apart from activating the immune system via non-specific processes, the BC can also trigger molecular recognition mechanisms (**Figure 4**).

This generally happens when the BC is enriched with opsonins, i.e. extracellular proteins that, when bound to foreign substances, make them more susceptible to the action of phagocytes and thus accelerate their degradation (187, 188, 203). Fibrinogen, immunoglobulins, and components of the complement system represent the most abundant opsonins found in the BC of NPs. Fibrinogen has a pivotal role in leukocyte activation and this process is notably intensified when fibrinogen adopts an unfolded state. Interestingly, unfolded fibrinogen in the BC of (PAA)-conjugated Au-NPs binds to the integrin receptor MAC-1, thus triggering an inflammatory response through the activation of the NF-κB signaling pathway (204, 205). This resulted in increased immunotoxicity of the systems. Furthermore, the BC has the capability to induce activation of the complement system, either by classical, alternative or lectin pathways. For example, Vu et al. elucidated that only a small number of surface-bound immunoglobulin molecules are required to initiate complement activation and opsonization (206). Tavano et al. demonstrated that NPs conjugated with poly(2-methyl-2-oxazoline), upon exposure to human serum, activate the classical complement pathway. In that work, C1q was identified as the initiating molecule capable of directly binding to NPs and promoting a rapid opsonization via the complement protein C3. Consequently, NPs were recognized and internalized by human polymorphonuclear granulocytes and monocyte-derived macrophages (207). In turn, dextran-coated superparamagnetic iron oxide core-shell nanoworms, when incubated in human serum and plasma, undergo rapid opsonization with the third complement component (C3) through the alternative pathway (208). Moreover, other pattern-recognition molecules have been recognized. Among these, mannose-binding lectin (MBL), ficolins and collectin detect various carbohydrate ligands on NPs and have been found to trigger the lectin pathway by activating MBL-associated serine proteases (MASPs) (209).

##### 2.2.4.2 Engineering the biomolecular corona to control the immune response

**Figure 5:**
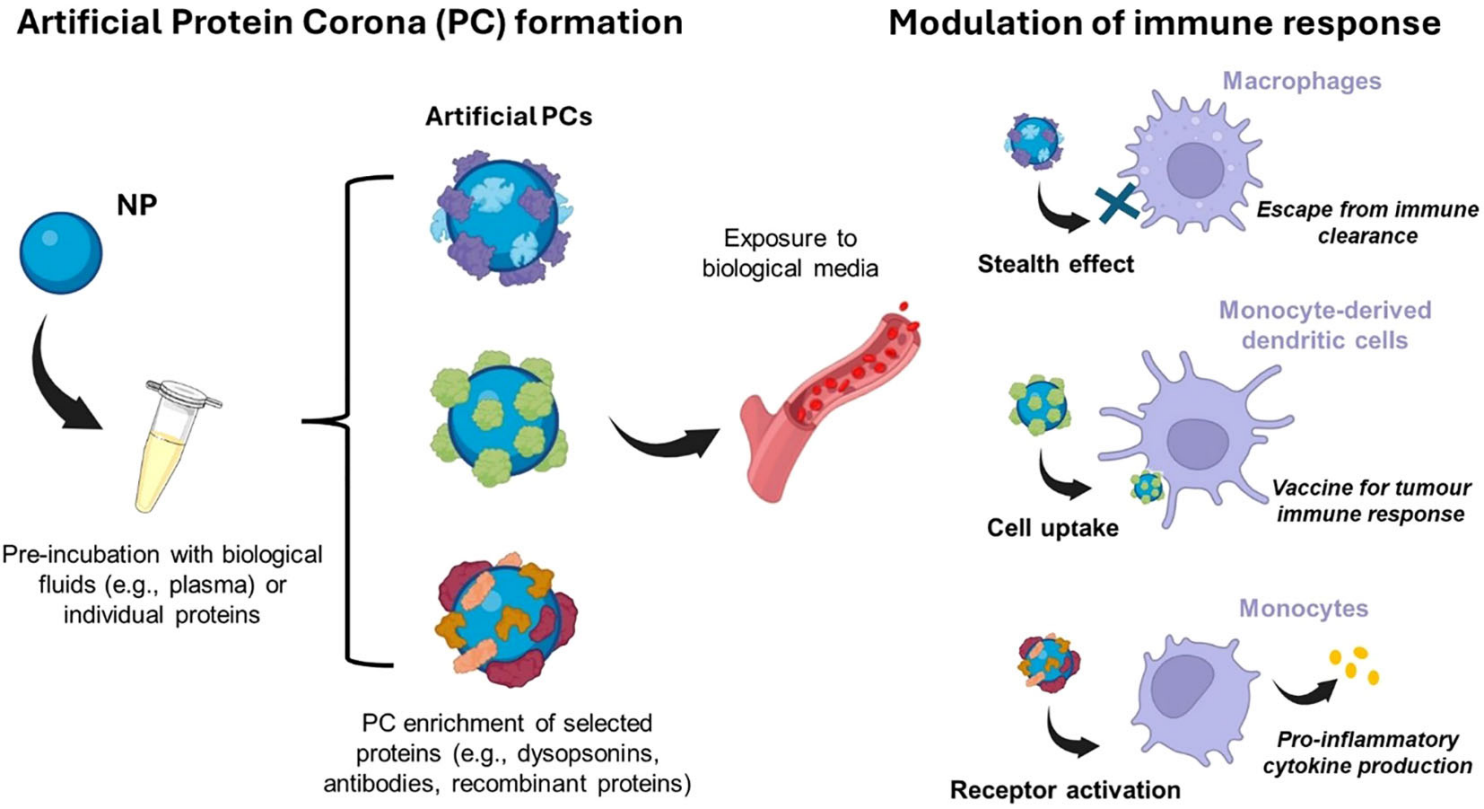
An emerging approach to modulate the immune system response is to pre-coat nanomaterials with an artificial protein corona (PC) before exposure to the biological environment. This artificial PC is generally exploited as a natural shield against immune system clearance or to activate immune cells for various purposes (e.g., pro-inflammatory cytokine production). Created with BioRender.com.

Since adsorbed proteins can induce complement processes that culminate in inflammation, nanomaterials should be engineered to mitigate such immune toxicity and enhance safety. Surface modification emerged as a possible strategy toward this goal. In this regard, grafting stealth components, e.g. polyethylene glycol (PEG), to the surface of NPs may enhance colloidal stability through steric repulsion (210), reduce aggregation, phagocytosis, and thus prolong systemic circulation (117). It is now firmly established that the formation of a BC is an unavoidable process for any nanomaterial, whether stealth-modified (e.g. PEG-functionalized) or not (188). The recognition of the BC as an inevitable aspect of NP interactions with biological systems, along with the potential benefits of proteins that improve NP pharmacokinetics, has stimulated interest in modifying the composition of the BC rather than developing materials that evade BC formation (211). Therefore, an emerging approach involves pre-coating nanomaterials with an artificial BC, providing a natural shield against immune cell clearance (**Figure 5**).

If the functional epitopes of corona proteins are not recognized by immune cell receptors, the BC itself could exhibit this shielding capacity. Artificial BC has been developed for brain targeting (212), and it involved the non-covalent functionalization of NPs with individual proteins. Recent studies have shown that non-covalent binding of the desired ligands is more efficient compared to other types of conjugation methods (213). In this context, Giulimondi et al. exploited the electric charge of DNA to create a lipid-based gene delivery system with a negatively charged surface that, upon exposure to human plasma, becomes coated with a BC enriched in dysopsonins, resulting in a biomimetic NP type with inherent stealth properties (214). In accordance, Tonigoldi et al., supported the beneficial of pre-coating NPs with artificial BC for enhancing NPs targeting ability and monitoring immune response (213). The authors selected the CD63 antibody, which binds to antigens on target cells such as monocyte-derived dendritic cells (moDCs), playing significant roles in the immune system. The study showed that NPs with covalently coupled antibodies lost their targeting ability, whereas those with pre-adsorbed antibodies remained highly functional in terms of targeting efficiency. Another study demonstrated that artificial coronas offer a means to precisely modulate the immune response, paving the way for innovative strategies in disease prevention and treatment (215). In particular, coating cationic liposomes with gelsolin, a key plasma protein involved in macrophage activation and modulation of inflammatory cytokine expression, triggers the activation of immune response in terms of uptake on innate immune THP-1 cells by modulating the pro-inflammatory cytokine production. On the other hand, gelsolin-coated DOTAP exposed to human plasma to mimic *in vivo* condition, showed a massive uptake by immune cells with phagocyte activity (i.e., monocytes and granulocytes) and retains the capability to induce tumor necrosis factor alpha (TNFα) production by monocytes. Yet, it was also determined that understanding the composition of the corona alone is insufficient to accurately predict immune cell capture. For precise prediction, it is essential to decipher the presentation of functional motifs at the interface. Dawson and colleagues developed effective methods for mapping protein binding sites on the BC using antibody-labeled gold NPs, differential centrifugal sedimentation, and imaging techniques (216). In line with this, Oh et al. engineered a nano-bio complex using recombinant proteins, forming a functional BC that served as a targeting moiety and a protein shield with minimal reactivity to other proteins when immersed in biological fluids. The NPs’ surface was pre-modified with a target linker capable of binding to a specific site on the recombinant protein. During the protein-NP interaction process, the recombinant proteins rapidly formed a stable protein layer on the NP surface. This layer hindered the clearance by the MPS facilitating the targeting to specific tumor tissue (217). Finally, activating immune cells by BC is emerging as a promising approach in innovative medical applications. Tumor-associated macrophages (TAMs) have been recognized as a crucial component of the tumor microenvironment. Pre-coating NPs with antibodies is a widely employed strategy for active targeting of TAMs (218). However, despite their specificity for certain cells and macrophages, their effectiveness is compromised by interactions with other cells expressing the same receptors and by significant nonspecific uptake by macrophages via Fc recognition. For instance, while CD206, a pattern recognition receptor, is upregulated on M2-like macrophages, it is also expressed by tissue-resident macrophages and dendritic cells. Although the mechanisms governing the interaction between TAMs and cancer cells remain incompletely understood, strategies employing modulation of BC for TAMs targeting to potentially restrain tumor advancement and eliminate metastases are still being explored (219). As an instance, it has been found that conformational alterations in proteins bound to nanomaterials can incite inflammatory reactions. Interactions with the surface of NPs drive alterations in the tertiary structure of proteins. These changes can uncover functional groups typically hidden within the hydrophobic core of the protein, promoting protein aggregation and amyloid fiber formation (220). Amyloid fiber formation, in turn, triggers inflammatory responses through receptor-mediated recognition, activating the immune cascade. Recently, the concept of an “amyloid BC” has also been exploited to develop novel sensing and mitigation strategies against amyloid-associated diseases, such as AD (220).

## 3 Discussion

The use of NPs to modulate immune responses within the CNS offers new exciting opportunities for therapeutic interventions and rehabilitation strategies for CNS disorders (9, 211). Researchers have explored various strategies to harness the unique properties of NPs for immune modulation, aiming to mitigate neuroinflammation, enhance tissue repair, and restore CNS homeostasis.

One promising approach involves the use of NPs as carriers for immunomodulatory agents, such as cytokines, antibodies, or small molecules, to selectively target and modulate specific immune cell populations within the CNS (8, 77). By encapsulating these agents within NPs, researchers can achieve controlled release kinetics, prolonged circulation times, and enhanced bioavailability, thus maximizing their therapeutic effects while minimizing systemic side effects (221, 222).

Additionally, NPs can serve as platforms for delivering nucleic acid-based therapeutics, such as small interfering RNA (siRNA) or messenger RNA (mRNA), to regulate gene expression and modulate immune responses within the CNS (10, 223). Through precise targeting and delivery, NPs enable efficient intracellular delivery of nucleic acids to immune cells, allowing for the selective suppression or activation of immune pathways implicated in CNS disorders.

Several studies have demonstrated the efficacy of NP-mediated immune modulation in preclinical models of neurological diseases, including multiple sclerosis, stroke, AD, and PD (224–232). For example, NPs coated with myelin-derived antigens have been shown to induce antigen-specific tolerance and suppress autoimmune responses in experimental models of multiple sclerosis, capable of inducing robust tolerance and long-term comprehensive disease protection, offering a promising approach for treating autoimmune neuroinflammatory disorders (233, 234).

Furthermore, advancements in NPs engineering and design have led to the development of multifunctional nanoplatforms capable of simultaneously targeting multiple immune pathways or delivering combination therapies within the CNS. These multifunctional NPs offer synergistic therapeutic effects and enhanced therapeutic outcomes compared to single-agent approaches, paving the way for personalized and precision medicine in CNS immunotherapy (235–238).

Another factor driving the development of nanotherapeutic strategies tailored to address the unique challenges of CNS diseases is the potential for targeted and precise drug delivery to specific cell types or regions within the CNS. The nano-bio interface with the immune system is key in the conceptualization of NPs to selectively target immune cells involved in neuroinflammation, such as microglia and astrocytes, while sparing healthy neurons. This targeted delivery approach minimizes off-target effects and systemic toxicity, enhancing the safety and efficacy of CNS therapeutics (76, 236, 239, 240).

Moreover, the biomolecular corona plays a pivotal role in dictating NP behavior and immune recognition within the CNS, giving researchers the opportunity to optimize NP design and surface modifications to minimize immunogenicity and enhance biocompatibility (211, 217, 219). By tailoring NPs to evade immune clearance and enhance cellular uptake, researchers can improve drug delivery efficiency and therapeutic outcomes in CNS disorders.

The significance of these advancements extends beyond drug delivery to encompass immune modulation, neuroprotection, personalized medicine, and precision therapeutics within the CNS. Tailored NPs are promising tools for rehabilitation and treatment of CNS conditions such as AD, PD and stroke (224, 232). NPs offer a versatile platform for delivering immunomodulatory agents and neuroprotective factors directly to diseased tissues, thereby modulating immune responses, reducing neuroinflammation, and promoting tissue repair. This immune reprogramming capability holds promise for treating a wide range of neurological conditions, including neurodegenerative diseases, autoimmune disorders, and brain tumors. With advances in nanotechnology, biomarker discovery, and imaging modalities, clinicians can tailor treatment regimens to the immune profiles and disease characteristics of individual patients (77, 236, 240).

In conclusion, the integration of NPs into CNS nanotherapeutics offers promising opportunities for addressing the complex challenges of neurological conditions and pathologies. By harnessing the nano-bio immune interface and understanding the significance of the biomolecular corona, researchers can develop targeted, safe, and effective nanotherapeutic interventions for a wide range of CNS disorders. These advancements have the potential to revolutionize the treatment landscape and rehabilitation of neurological diseases, offering hope for improved patient care and quality of life in the future.

### 3.1 Future directions and implications

While there have been many advancements in the field, several key avenues emerge for future research aimed at optimizing immune-NP interactions and harnessing their potential for enhanced CNS therapeutics. One promising direction involves further elucidating the dynamic interplay between NPs and the immune system within the CNS microenvironment. Moreover, there is a pressing need to explore the therapeutic potential of immune-NP interactions in specific CNS disorders, including neuroinflammatory diseases, neurodegenerative disorders, and cancers. By tailoring NP properties to modulate immune responses associated with these conditions, researchers can develop personalized nanotherapeutic interventions that address the contextual pathophysiology and improve patient outcomes (217, 218).

Beyond the realm of CNS nanotherapeutics, understanding the nano-bio immune interface holds broader implications for the development of novel nanotherapeutic strategies across various disease contexts. By deciphering the principles governing immune-NP interactions, researchers can apply this knowledge to improve rehabilitation and design innovative and personalized nanomedicines for targeting systemic immune disorders, infectious diseases, and cancer.
